# Transcriptomic Changes in Response to Form of Selenium on the Interferon-Tau Signaling Mechanism in the Caruncular Tissue of Beef Heifers at Maternal Recognition of Pregnancy

**DOI:** 10.3390/ijms242417327

**Published:** 2023-12-10

**Authors:** Sarah N. Carr, Benjamin R. Crites, Harshraj Shinde, Phillip J. Bridges

**Affiliations:** Department of Animal and Food Sciences, University of Kentucky, Lexington, KY 40546, USA; sarah.carr@uky.edu (S.N.C.); benjamin.crites@uky.edu (B.R.C.); harshraj19@uky.edu (H.S.)

**Keywords:** selenium, selenoproteins, caruncle, endometrium, maternal recognition of pregnancy, interferon tau, interferon signaling, beef cattle

## Abstract

We have reported that selenium (Se) provided to grazing beef cattle in an inorganic (ISe) form versus a 1:1 mixture (MIX) of inorganic and organic (OSe) forms affects cholesterol biosynthesis in the corpus luteum (CL), the abundance of interferon tau (IFNτ) and progesterone (P4)-induced mRNAs in the caruncular (CAR) tissue of the endometrium, and conceptus length at maternal recognition of pregnancy (MRP). In this study, beef heifers were supplemented with a vitamin–mineral mix containing 35 ppm Se as ISe or MIX to achieve a Se-adequate status. Inseminated heifers were killed at MRP (d 17, n = 6 per treatment) for tissue collection. In CAR samples from MIX versus ISe heifers, qPCR revealed that mRNA encoding the thyroid regulating DIO2 and DIO3 was decreased (*p* < 0.05) and a complete transcriptomic analysis revealed effects on the interferon JAK-STAT1/2 pathway, including decreased expression of mRNAs encoding the classical interferon stimulated genes IFIT1, IFIT2, IFIT3, IRF1, IRF9, ISG15, OAS2, and RSAD2 (*p* < 0.05). Treatment also affected the abundance of mRNAs contributing to the immunotolerant environment (*p* < 0.05). In combination, these findings suggest more advanced preparation of the CAR and developing conceptus for implantation and to evade immune rejection by the maternal system in MIX- vs. ISe-treated heifers.

## 1. Introduction

Selenium (Se) was initially recognized as having toxic effects on both humans and livestock [1], with the critical role of this mineral defined within the subsequent years [2,3,4]. This trace mineral is an essential micronutrient at low levels, but is toxic at high levels [5], providing a narrow aperture between dietary adequacy and toxicity [6]. The physiological relevance of Se was initially identified as a necessary structural component of glutathione peroxidases (GPX; [7]), a group of selenoproteins with antioxidant activities that catalyze harmful hydrogen peroxide (H_2_O_2_) into water (H_2_O [8]). Presently, there are 25 selenoprotein genes identified in humans [8] and pigs [9], and 25 selenoproteins that we routinely investigate in cattle [10,11]. Many selenoproteins, including selenoprotein H (SELENOH [12]), selenoprotein K (SELENOK [13]), selenoprotein M (SELENOM [14]), selenoprotein P (SELENOP [15]), selenoprotein R (SELENOR [16]), and selenoprotein W (SELENOW [17]), have been identified or proposed to have antioxidant capabilities. Harmful oxidative species can cause damage to DNA, RNA, and proteins, and can engender cell death [18]. Therefore, it is necessary for the cells to effectively scavenge reactive oxygen species (ROS) to protect cells from oxidative stress.

Across the United States, the concentration of Se in the soils and, subsequently, in the forages available to grazing livestock varies by geographic region [19]. The concentration of Se in the forages and grains in the southeastern United States, including Kentucky, are low, at less than 0.05 ppm, to variable. Only about 50% of the feedstuffs produced contain greater than 0.1 ppm [19], the minimal recommended dietary intake of this trace mineral [20]. This provides a challenge for cattle producers in these Se-deficient regions; hence, it is necessary to supplement Se in the diet of cattle grazing these forages [21,22,23,24,25]. Providing this auxiliary Se to overcome a deficiency in this trace mineral is essential for various physiological processes, including immune function [24,26], growth [22], antioxidant status [27], and fertility [23]. Commonly, supplemental Se is provided in a vitamin–mineral mix as an inorganic form (ISe), sodium selenite, or sodium selenate. However, organic forms of selenium (OSe), and particularly selenomethionine, are available when cattle graze forage [19].

We consistently studied the effects of form of supplemental Se as either ISe or a 50%:50% mixture of ISe to OSe (MIX) on gonadal function in cattle and reported a MIX-induced increase in the systemic concentration of progesterone (P4) in the early luteal phase of the estrous cycle [28,29], with this increase occurring at a time that can significantly improve embryonic development by altering endometrial function that supports conceptus elongation [30,31,32] and the production of interferon tau (IFNτ), a protein from the developing conceptus that signals maternal recognition of pregnancy (MRP [33]). Indeed, we observed a significant increase in the length of the conceptus in MIX versus ISe-supplemented heifers, which has clear implications for fertility outcomes as it may be better prepared for the impending processes of attachment and implantation [34]. Receptivity of the endometrium to allow implantation of the conceptus is dependent on P4 [35], uterine restructuring to promote placentation [36], and the ability of the fetal allograft to evade the maternal immune response [37]. Considerably, we performed a targeted qPCR analysis, determining changes in the relative abundance of several P4 and IFNτ-regulated mRNA transcripts in the caruncular (CAR) tissues of the endometrium from ISe- versus MIX-supplemented heifers at MRP (d 17 of gestation) and observed a significant decrease in the expression of mRNAs encoding diacylglycerol o-acyltransferase 2 (DGAT2), fibroblast growth factor 2 (FGF2), interferon induced protein with tetratricopeptide repeats 3 (IFIT3), ISG15 ubiquitin like modifier (ISG15), MX dynamin like GTPase 1 (MX1), 2′-5′-oligoadenylate synthetase 2 (OAS2), and radical S-adenosyl methionine domain containing 2 (RSAD2 [34]), with this study expanding on those results.

The necessary immunotolerant environment is the result of a careful balance between compositional changes in the innate immune system, which appears to remain active to protect against pathogens [38], and the adaptive immune system, which must be repressed [37,38]. During this time period, ligands from the conceptus signal the endometrium to effect necessary changes, and in the presence of P4, there is an increase in macrophages, dendritic cells, and NK cells that migrate into uterine epithelial, endothelial, and stomal cells [37], as well as changes in the abundance of molecules that promote immune tolerance such as indoleamine 2,3-dioxygenase 1 (IDO1), transforming growth factor beta (TGFB), and interferon stimulated genes (ISG) [37].

In our present experiment, commercial Angus-cross heifers (N = 20) received 45-day ad libitum access to a basal vitamin–mineral mix with no exogenous source of Se (depletion phase), followed by 45-day supplementation with a vitamin–mineral mix containing 35 ppm Se as ISe (repletion phase), and then were randomly assigned to received ad libitum access to supplemental treatment as either inorganic (ISe; sodium selenite, n = 10) or a 1:1 combination (MIX, n = 10) of ISe and OSe (1:1 sodium selenite and SEL-PLEX) for at least 90 days prior to estrous synchronization and artificial insemination. Heifers were then killed at d 17 of presumed pregnancy. An intact conceptus and other experimental samples were collected from both ISe- and MIX-treated heifers (n = 6 per treatment) immediately after death. Caruncular (CAR) tissue was used herein; however, the embryo, CAR and intercaruncular (ICAR) tissues, whole blood, and serum were utilized in Crites et al. [34].

This experiment had two primary objectives. Firstly, we aimed to quantify the changes in mRNA transcripts of selenoproteins with known or proposed antioxidant capabilities and select selenoprotein receptors in response to the form of Se at MRP. Our hypothesis was that a cohort of these enzymes, and particularly the cytosolic GPX1, will be of greater abundance in MIX- vs. ISe-treated heifers, which would correspond with the whole animal’s ability to mitigate the increased ROS that is associated with an increased metabolic demand at MRP. Subsequently, with the absence of a global perspective on the effect of form of Se on mRNA transcripts in the caruncular regions of the endometrium, the second objective of this study was to examine transcriptomic changes in the CAR tissue at MRP, postulating that we would observe MIX-induced changes in transcript expression indicating advancement of the CAR transcriptome in preparation for the impending processes of attachment and implantation. Defining the mRNA profile of selenoproteins and the transcriptome of CAR tissue as a whole will provide insight into how the form of Se is related to improved fertility and longer conceptuses at MRP. Ultimately, this provides a producer-friendly and sustainable method to increase whole farm productivity and profitability of grazing beef cattle.

## 2. Results

### 2.1. Real-Time PCR Analysis of Selenoproteins and Selenoprotein P Receptors mRNA

A total of twenty-nine selenoprotein and selenoprotein P receptor transcripts were targeted via qPCR analysis. The MIX form of supplemental Se significantly (*p* < 0.05) decreased the relative abundance of *Dio2* and *Dio3* mRNAs (Table 1). The relative abundance of transcripts encoding the other targeted selenoproteins and the selenoprotein P receptors was not affected by form of Se. RNAseq also indicated that the MIX form of Se significantly (*p* < 0.05) decreased the relative abundance of *Txnrd2* and *Selenon* mRNAs, and increased the abundance of *Selnok* mRNA; however, these findings were not confirmed by qPCR.

### 2.2. Cluster Analyses

**Figure 1:**
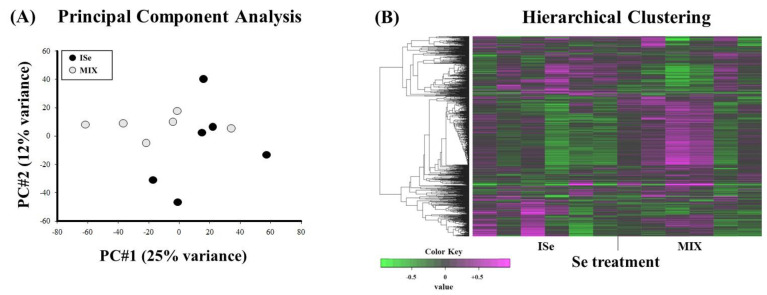
(**A**) Score plot and (**B**) hierarchical matrix of data derived from RNA-Seq analysis of each sample of caruncular (CAR) tissue from heifers supplemented with a vitamin–mineral mix containing 35 ppm as ISe (n = 6) or 1:1 mixture of ISe:OSe (MIX, n = 6). (**A**) Principal component 1 (PC#1) accounted for 25% of the variance, and principal component 2 (PC#2) accounted for 12% of the variation among samples. (**B**) Hierarchical cluster analysis was composed of the top 1000 differentially expressed transcripts using iDEP.96. Significance was determined by Wald test, with significance declared at *p* < 0.05 for all.

Principal component analysis (PCA) of all RNA-Seq data was performed to evaluate the relative relationships and variation among the individual heifers. The score plot (Figure 1A) demonstrates that principal component 1 (PC#1, x-axis) explained 25% of variance among the samples and principal component 2 (PC#2, y-axis) explained 12% of variance. Results indicate that there is slight separation between treatment groups, with some similarities in the expression of DEGs.

Hierarchical clustering analysis of the top 1000 DEGs (Figure 1B) indicates a considerable separation between Se treatments; however, there may be some overlap in transcriptomic profiles of the CAR from ISe- and MIX-supplemented heifers.

### 2.3. Differentially Expressed Genes

The Wald test of DESeq2 was used to identify changes in CAR between the ISe- and MIX-supplemented heifers. At *p* < 0.05, there were 2029 DEGs with 1038 transcripts upregulated, and 991 transcripts downregulated in MIX compared to ISe. The most differentiated genes that were increased and decreased according to fold change in MIX compared to ISe are provided in Table 2.

### 2.4. Pathway and Gene Network Analysis

**Figure 2:**
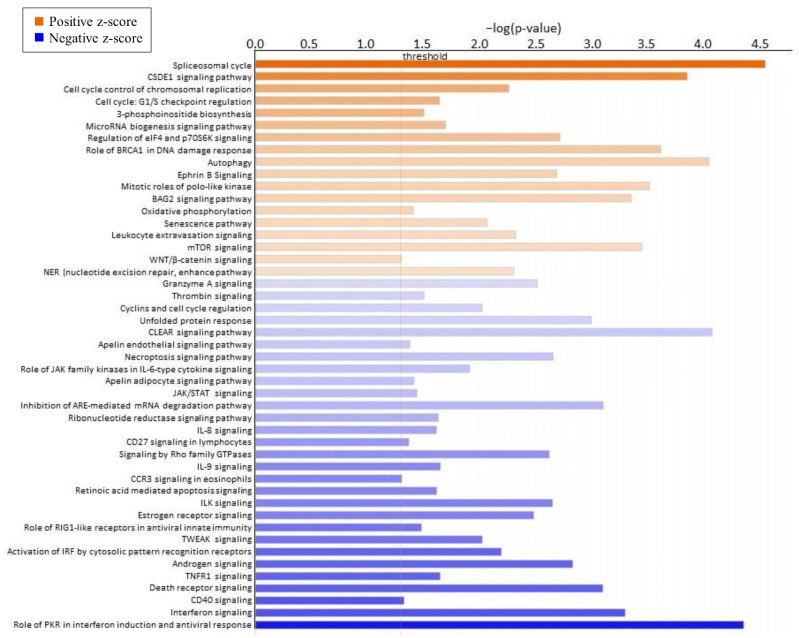
Top IPA-identified canonical pathways of genes differentially expressed from CAR of heifers supplemented a vitamin–mineral mix containing 35 ppm as ISe (n = 6) or 1:1 mixture of ISe:OSe (MIX, n = 6). Pathways are ranked by z-score, with orange indicating a pathway that is predicted to be upregulated (positive z-score) and the blue indicating a pathway that is predicted to be downregulated (negative z-score).

To determine global changes in DEGs in response to form of Se, bioinformatic analysis was performed using QIAGEN’s Ingenuity Pathway Analysis (IPA, QIAGEN, Redwood City, CA, USA). Canonical pathway analysis indicated that the top five canonical pathways (Table 3) based on *p*-values affected by the form of Se are ATM signaling (*p* < 0.0001), spliceosomal cycle (*p* < 0.0001), role of protein kinase R (PKR) in interferon induction and antiviral response (*p* < 0.0001), coordinated lysosomal expression and regulation (CLEAR) signaling pathway (*p* < 0.0001), and autophagy (*p* < 0.0001). Further, Figure 2 shows the top canonical pathways in CAR ranked by z-score, which is used to predict activation state. Interestingly, the most significantly affected pathways that were positively affected are spliceosomal cycle (z-score = 2.673), cold shock domain containing E1 (CSDE1) signaling pathway (z-score = 2.138), cell cycle control of chromosomal replication (z-score = 1.508), cell cycle: G1/S checkpoint regulation (z-score = 1.414), and 3-phosphoinositide biosynthesis (z-score = 1.342). The most negatively affected pathways based on z-score are the role of PKR in interferon induction and antiviral response (z-score = −3.128), interferon signaling (z-score = −2.333), CD40 signaling (z-score = −2.333), death receptor signaling (z-score = −2.183), and TNF receptor superfamily member 1A (TNFR1) signaling (z-score = −2.121).

The top upstream regulators identified using IPA at *p* < 0.05 were hepatocyte nuclear factor 4 alpha (HNF4A), estrogen receptor 1 (ESR1), KRAS proto-oncogene, GTPase (KRAS), transforming growth factor beta 1 (TGFB1), and Pyridostatin. The top five molecular and cellular functions with specific actions identified by IPA are indicated in Table 4. Only functions with a z-score greater than or equal to the absolute value of 0.5 are reported.

### 2.5. Real-Time PCR Analysis of Select mRNA Transcripts

To validate the outcomes of the RNA-sequencing, real-time PCR analysis was conducted on select transcripts that are responsive to interferons or are relative to uterine function at MRP (Table 5). The results of qPCR were consistent with RNA-sequencing analysis at either significance, declared at *p* ≤ 0.05, or a tendency at 0.05 < *p* ≤ 0.10, except for *Irf9*, *Isg20,* and *Scarb1* mRNA. However, these were consistent in trend when compared to RNA-sequencing results, but were not significant for qPCR analyses. We did not corroborate all identified RNA-sequencing transcripts of interest; some are included below for purposes of clarity in the discussion.

## 3. Discussion

Soils and hence forages in geographic regions that are low in Se require producers to supplement this trace mineral in the diet of beef cattle to overcome Se-deficiency-associated challenges with immune function [24,26], growth [22], antioxidant status [27], and fertility [23]. Commonly, supplemental Se is provided in a vitamin–mineral mix as ISe; however, Ose forms are available when cattle graze forage [19].

The organic forms of Se are more bioavailable than the inorganic forms [39], and organic selenium can raise whole blood levels of Se more effectively compared to the inorganic sodium selenite or sodium selenate [40]. Additionally, transcriptomic analysis in the liver of growing beef heifers supplemented with Se as either ISe, OSe, or a 50%:50% mixture of ISe or OSe (MIX) demonstrated that the form of Se creates specific phenotypes respective to form, with distinct physiological capabilities including changes in redox potential. Additionally, these transcriptomic changes were not indicative of a gradient effect respective to the different forms of Se utilized [41].

Using an extensively reported model [10,28,29,42,43], we studied the effects of supplemental form of Se as ISe (the industry standard) or a 1:1 mixture of ISe to OSe (MIX) on grazing beef cows and heifers and observed significant effects on the CL [10] and endometrium [34], which laid the premise for the present study. This experiment had two specific objectives. Firstly, we aimed to quantify the changes in mRNA transcripts of selenoproteins with known or proposed antioxidant capabilities and select selenoprotein P receptors in caruncular (CAR) tissue in response to form of Se at MRP. Secondly, this study sought to augment previous findings in our lab that identified key caruncular changes in the abundance of mRNA encoding several P4 and IFNτ-induced endometrial transcripts, which occurred synonymously with longer conceptuses in MIX- versus ISe-supplemented heifers at MRP [34]. Therefore, we performed a transcriptomic analysis to elucidate global changes in the CAR transcriptome in response to form of Se. These results expand our understanding of mechanistic changes in the CAR that may be contributing to the previously observed significant differences in conceptus length at MRP and may be better preparing the CAR for attachment and implantation.

### 3.1. Selenoproteins

There are 25 selenoprotein genes identified in mammals [8,9], and we sought to analyze the Se-form effects on mRNA transcripts, encoding these, as well as select selenoprotein P receptors, in the caruncular tissue of heifers at MRP (d 17 of gestation). We hypothesized that a cohort of these enzymes, and particularly the cytosolic GPX1, will be of greater abundance in MIX- vs. ISe-treated heifers, which would indicate changes in the ability to mitigate potentially elevated reactive oxygen species associated with an increased metabolic demand at MRP, with the CAR at this time differentiating in early preparation for implantation and placentation.

Unexpectedly, we only observed changes in the abundance of mRNA transcripts encoding the intracellular iodothyronine deiodinases 2 and 3 *(Dio2* and *Dio3),* and the relative abundance of mRNA encoding these two selenoproteins was significantly lower (*p* < 0.05) in MIX-supplemented heifers compared to those on ISe alone. Iodothyronine deiodinases (DIOs) are a family of selenoproteins that regulate the activation and deactivation of thyroid hormones, and, subsequently, growth, development, thermogenesis, cell differentiation and proliferation, and energy metabolism, including regulating metabolism of carbohydrates, proteins, and lipids [44,45]. Regulating the circulating and intracellular concentrations of thyroid hormones is critical for placental development and function, as well as fetal growth [45].

DIO1 is bound in the plasma membrane and has been primarily localized to the liver, kidney, and thyroid [8]. This selenoprotein converts T4 to T3 by deiodination of the outer ring, and it can convert T3 and T4 into the inactive T2 or rT3, respectively, via inner ring deiodination [46]. Given the primary localization and function of DIO1, it is not unsurprising that we were unable to detect a measurable concentration of mRNA encoding this protein in CAR samples; however, it should be noted that previous studies have shown DIO1 to be abundant in the term placenta [47], which may be associated with circulating concentrations of T3 being highest at parturition [47,48].

Intracellular DIO2 is located in the endoplasmic reticulum and is primarily expressed in the nervous system, pituitary, thyroid, and brown adipose tissue [8]. Low levels of serum T4 can result in an elevation in the activity of DIO2 [49]. DIO3 is another membrane-bound selenoprotein that is primarily expressed in vascular tissue, skin, and the placenta, as well as being highly expressed in fetal and neonatal tissues [44]. Its high level of expression in the placenta is to protect the growing fetus from the activity of maternal thyroid hormone as DIO3 primarily inactivates T3 [50]. However, in ruminants, the placenta may be impermeable to transfer of thyroid hormones [45]. It seems plausible that T3 could be effectively regulating the structural and metabolic changes occurring in the CAR in preparation for placental development in our experimental animals, which would be consistent with the previously observed longer embryos in MIX- versus ISe-supplemented heifers at MRP [34].

### 3.2. Caruncular Transcriptomics

With the absence of a global perspective of mRNA transcripts in the CAR in response to form of Se, we performed a transcriptomic analysis using next-generation sequencing to reveal the form of Se-affected pathways that may advance or impede the impending placental attachment. Our previous observation that MIX-supplemented heifers had significantly longer conceptuses on d 17 of gestation, coincident with a decreased level of expression of known P4- and INF-induced transcripts, including DGAT2, MX1, and OAS2 in the CAR [34], suggests that the process of MRP is more advanced in these heifers, as may be the development of the caruncular transcriptome in preparation for the initial process of adhesion and attachment, which is observed as early as days 16–18 of pregnancy in cattle [51,52]. Results from the current transcriptomic analysis correspond with this, as evidenced by the significant changes in both interferon signaling, which is affecting the transcription of interferon responsive genes, and by the noted changes in transcripts regulating cellular organization, survival, and death.

Prominently, the top two canonical pathways downregulated in MIX-treated heifers were associated with endometrial response to type I interferon signaling (“Role of PKR in interferon induction and antiviral response” and “Interferon signaling”). The trophoblast-derived IFNτ is a type I interferon that signals by binding to the ubiquitously expressed type 1 interferon receptors (IFNAR1 and IFNAR2) on the endometrial luminal and superficial glandular epithelium [38,53]. The primary function of this signal is to inhibit transcription of the estrogen (ER), effectively preventing estrogen-induced increases in the synthesis of ER, nuclear progesterone receptor (PGR), and oxytocin receptor (OTR), thus blocking production of luteolytic pulses of prostaglandin F_2α_ (PGF_2α_). This mechanism also appears to involve the actions of IRF2 [30]. Presently, and reported in Crites et al. [34], the presence of a more developed conceptus in MIX- versus ISe-treated heifers demonstrated successful signaling at MRP. However, the absence of Se-form effects on the abundance of mRNA encoding IFNAR1, IFNAR2, IRF2, ER, or OXTR suggests that there are mechanisms still to be elucidated that may be of significance. Additionally, as reported in Crites et al. [34], qPCR analysis revealed a tendency for *Pgr* mRNA to be less abundant in MIX, which is consistent with the previously observed decrease in the expression of *Pgr* in response to continuous exposure of P4 in the endometrium [54].

**Figure 3:**
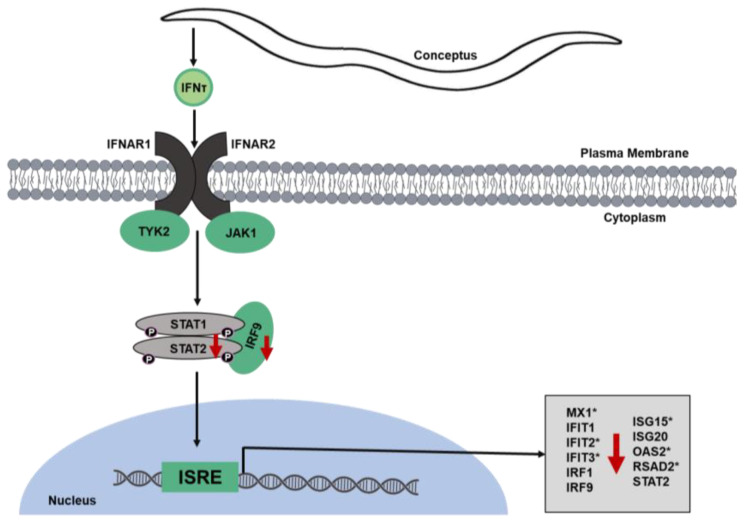
Mechanistic illustration of downregulation of interferon-responsive genes and JAK/STAT1/2 signaling pathway. Red arrows demonstrate lower transcript abundance in MIX-Se- compared to ISe-supplemented heifers. All transcripts included were significant via transcriptomic analysis, and an asterisk (*) indicates those transcripts that were corroborated via qPCR at *p ≤* 0.05. Results of qPCR analysis for MX1 are reported in Crites et al. [34].

In conjunction with being antiluteolytic, IFNτ exerts immense effects in the endometrium during MRP by promoting the transcription of interferon stimulated genes (ISGs, [55,56]). To do so, IFNτ binds IFNAR1 and IFNAR2 to activate the JAK/STAT signal transduction pathway. STAT1 can form a homodimer that translocates into the nucleus to stimulate the transcription of specific genes by binding to interferon-gamma-activated sequence (GAS). Alternatively, STAT1 and STAT2 form a heterodimer and interact with IRF9 to form the transcriptionally active gene complex factor 3. This complex migrates into the nucleus to bind interferon stimulated response elements (ISRE) that facilitate transcription of ISGs (Figure 3) [38,56,57].

Results of the present transcriptomic analysis revealed significant MIX-form downregulation in the abundance of ISGs, particularly to those that are activated by ISRE. During RNA-sequencing analysis, we observed a significant MIX-induced decrease in *Stat2* and *Irf9* mRNA with no change in mRNA encoding STAT1. This suggests that downregulation of ISG is occurring via the interferon gene complex factor 3 protein complex that translocates into the nucleus to bind ISRE in the promoter regions of ISGs [38]. Additionally, STAT2 and IRF9 self-regulate transcription of their own genes, thus leading to potentially further effects on the type 1 interferon JAK/STAT signaling transduction pathway [58].

Of the classical ISGs that are transcribed following activation of ISRE, mRNA transcripts for IFIT2, IFIT3, IRF9, ISG15, and STAT2 were significantly downregulated in MIX-supplemented heifers (Figure 3). We further detected significant decreases in mRNAs encoding the interferon-stimulated genes OAS2, RSAD2 and ISG20, and we previously reported a MIX-Se downregulation of *Mx1* mRNA at this time [34].

Results support the present concept that the peak signal of IFNτ from the developing conceptus occurred earlier in MIX supplemented heifers, as there was a stark decrease in the abundance of mRNA transcripts encoding IRF9. Notably, *Irf9* mRNA is typically more abundant between days 14–18 compared to days 25–40 of pregnancy in cattle, coincident with decreases in mRNA encoding IRF3, MX1α, MX1β, and MX2 in CAR at these two time points [59]. To corroborate these findings, quantifying IFNτ in the uterine fluid would provide an indication of the concentration of this protein in the histotroph. Unfortunately, in the present study, recovery of the uterine fluid at sample collection proved too inconsistent for subsequent quantification of the concentrations of IFNτ protein.

For successful attachment and implantation to occur, there must also be restructuring of the endometrium and evasion of the maternal immune system by the developing conceptus [36,37]. Functional analysis of the significantly affected mRNA transcripts in the present study identified significant changes in the “spliceosomal cycle”, “cell death and survival”, “cellular assembly and organization”, and “autophagy” pathways, suggesting that significant structural and functional changes are occurring. The canonical pathway most predicted to be upregulated in MIX was the spliceosomal cycle. Most genes are transcribed as pre-mRNAs containing introns that must be spliced out to form the mRNA for translation [60,61,62]. Splicing of pre-mRNA is facilitated by the enzymatic activity of the spliceosome, consisting of five ribonucleoprotein subunits (snRNPs) and various protein co-factors [63,64]. This cycle is dependent on ATP [63] and is heavily regulated to ensure accurate splicing [60]. Although the spliceosome cycle is significantly affected by the form of supplemental Se, the physiological relevance of this effect is unclear at this time.

Effective attachment and implantation also require a transient evasion of the maternal immune system to allow the fetal allograft to closely interact with the maternal interface [38]. The control of this mechanism appears to be modulated predominantly by the endometrium, although it may also be stimulated by IFNτ and other signals from the developing conceptus [37,65]. This immunotolerant environment is the result of a careful balance between compositional changes in the innate immune system consisting of granulocytes, monocytes, and dendritic cells, which may remain constitutively responsive to protect against pathogens [38], and the adaptive immune system, which must be repressed so as to not reject the invading conceptus [37,38]. Ligands from the conceptus as well as prostaglandins act on the endometrium that has been primed by P4 to effect changes in both the innate and adaptive immune responses. There is a significant increase in the abundance of the MHC II-expressing molecules, macrophages, and dendritic cells, as well as NK cells that migrate into the uterine epithelial, endothelial, and stomal cells in the presence of elevated levels of P4. Additionally, there is an increased abundance of molecules that promote immune tolerance such as indoleamine 2,3-dioxygenase 1 (IDO1) and, subsequently, kynurenine, IL10, TGFB, and various cell surface receptors (CTLA4, PD-L1, and LAG3). Furthermore, there is increased expression of several ISGs associated with immune tolerance in the uterus and circulating immune cells [37].

Of interest, IDO1 catalyzes the rate-limiting step in the catabolism of tryptophan via the kynurenine pathway, although it is also critical in immune tolerance and protecting the embryo from rejection [37,66]. The relative abundance of this transcript was significantly reduced in MIX versus ISe CAR samples. When comparing pregnancy to cyclic cows, IDO1 is significantly more abundant on day 17 of gestation [66,67], but then dramatically declines by day 20 [67]. This elevated expression of IDO1 is coupled with a decrease in the relative concentration of tryptophan: kynurenine [66], and kynurenine can further affect immune tolerance [68]. Relatedly, mRNA encoding ISG20 and SCARA1 were significantly lower, and mRNA encoding the transforming growth factor beta 1 (TGFB1) tended to be lower in the MIX-supplemented heifers, which is indicative of a decreased antiviral response and an increased tolerance for cell invasion [37,38].

In combination, these findings suggest more advanced preparation of the developing conceptus to evade immune rejection by the maternal endometrium. This supports our hypothesis that there is a shift in the timing of peak IFNτ signaling (MRP), and advancement of the conceptus and endometrium to better support impending attachment, implantation, and placentation.

## 4. Materials and Methods

This project (protocol number 2017-2828) was approved by the University of Kentucky’s Institutional Animal Care and Use Committee.

### 4.1. Animals and Experimental Procedure

Angus-cross heifers (N = 20) received 45 days of Se depletion with no supplemental Se, followed by 45 days of Se repletion with a vitamin–mineral mix containing 35-ppm Se as only ISe to reestablish systemic Se to an adequate status [69,70]. The heifers were then randomly assigned to supplemental Se treatment formulated with a vitamin–mineral mix containing 35 ppm Se as either inorganic Se (n = 10, ISe, sodium selenite, Prince Agri Products, Inc., Quincy, IL, USA) or a 1:1 ratio of ISe and OSe (n = 10, MIX, SEL-PLEX; Alltech, Inc., Nicholasville, KY, USA). Heifers received dietary treatments for at least 90 days prior to estrous synchronization.

Whole blood was collected via jugular venipuncture prior to depletion, repletion, and the start of treatment. It was also recovered bimonthly during treatment until the end of experimentation to confirm Se adequate status throughout. Whole blood was also collected from each heifer at estrus and prior to slaughter (d 17). Total blood Se was quantified by the University of Kentucky’s Veterinary Diagnostics Laboratory (Lexington, KY, USA) using an Agilent 7900 inductively coupled plasma–mass spectrometer [71]. Throughout experimentation, all animals maintained Se-adequate status [69,70], and there tended to be an effect of Se treatment (*p* = 0.07) as previously described in Crites et al. [34].

### 4.2. Experimental Regimen and Serum Collection

After at least 90 days of Se supplementation with each respective treatment (ISe vs. MIX), heifers were randomly injected with one or two doses of dinoprost tromethamine (25 mg, Lutalyse, Zoetis, Parsippany, NJ, USA) to induce regression of the CL. Heifers were then monitored daily for behavioral estrus (d 0) by visual observation as well as using CowManager technology (Gerverscop 9, Harmelen, The Netherlands) to predict the timing of estrus. The presence of a preovulatory follicle (estrus, d 0) was confirmed via transrectal ultrasonography using a 5–8 MHz linear transducer (Ibex Pro, E.I. Medical Imaging, Loveland, CO, USA) prior to artificial insemination at 0 h, 12 h, and 24 h using commercially available frozen semen from a single established high-fertility bull.

The reproductive tract (ovaries, uterus, and cervix) was collected from each heifer on d 17 following euthanasia via captive bolt stunning and exsanguination at the USDA inspected University of Kentucky Meat Laboratory. Initially, the uterus was flushed to collect the conceptus as described in Crites et al. [34]. An intact conceptus was recovered from six heifers in each treatment group (ISe, n = 6; MIX, n = 6), and only these heifers were used for analyses in the present study.

Subsequently, caruncular endometrial samples were collected from the uterine horn ipsilateral to the ovary bearing the CL. This respective uterine horn was cut longitudinally to expose the uterine lumen, and an 8 mm biopsy punch (Integra LifeSciences Production Corporation, Mansfield, MA, USA) was used to collect CAR endometrial samples from all animals. These samples were flash-frozen in liquid N_2_ and stored at −80 °C to be used for RNA extraction, RNA sequencing, and for real-time PCR (qPCR) analysis.

### 4.3. RNA Extraction

Total RNA was extracted from approximately 200 mg CAR samples of the endometrium using TRIzol Reagent (Invitrogen Corporation, Carlsbad, CA, USA). All samples had high purity, with 260/280 absorbance ratios of 1.88 or greater as quantified using a NanoDrop ND-100 Spectrophotometer (NanoDrop Technologies, Wilmington, DE, USA).

### 4.4. RNA-Sequencing

Transcriptomic analysis using RNA-sequencing was conducted using mRNA extracted from CAR tissue. Library preparation was performed by Zymo Research Corporation (Irvine, CA, USA). Initially, 500 ng of total RNA was used to construct the total RNA-Seq libraries. The method described in Bogdanova et al. [72] was used to remove ribosomal RNA (rRNA) with some modifications, and libraries were prepared using the Zymo-Seq RiboFree Total RNA Library Prep Kit (Zymo Research Corporation, Irvine, CA, USA). The RNA-Seq libraries were then sequenced using an Illumina NovaSeq with a sequencing depth of at least 30 million read pairs per sample.

The RNA-Seq pipeline utilized by the Zymo Research Corporation (Irvine, CA, USA) was adapted from nf-core/rnaseq pipeline v1.4.2 [73] and built using Nextflow [74]. The quality of raw reads was analyzed using FastQC v0.11.9, and adaptor and low-quality reads were trimmed using Trim Galore! v0.6.6. The resultant trimmed reads were aligned to the reference genome using STAR v2.6.1d [75]. SAMtools v1.9 was used for BAM file filtering and indexing [76], and library quality control was executed using QualiMap v2.2.2-dev [77] and RSeQC v4.0.0 [78]. Duplicated reads were marked using Picard tools v2.23.9 (Broad Institute, Cambridge, MA, USA), and quality control of the duplication rate was analyzed using dupRadar v1.18.0 [79]. The complexity of the library was estimated using Preseq v2.0.3 [80], and gene assignments were applied to reads that overlapped with exons using featureCounts v2.0.1 [81].

**Figure 4:**
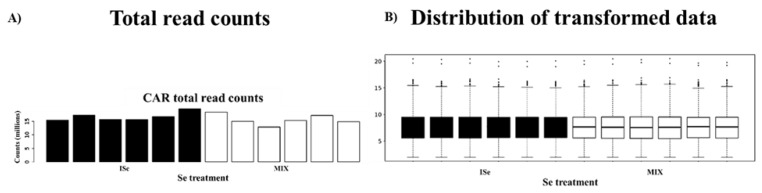
The (**A**) total read counts and (**B**) distribution of log2 transformed data derived from RNA-Seq analysis of each sample of caruncular (CAR) tissue from heifers supplemented with a vitamin–mineral mix containing 35 ppm as ISe (n = 6) or 1:1 mixture of ISe:OSe (MIX, n = 6).

**Figure 5:**
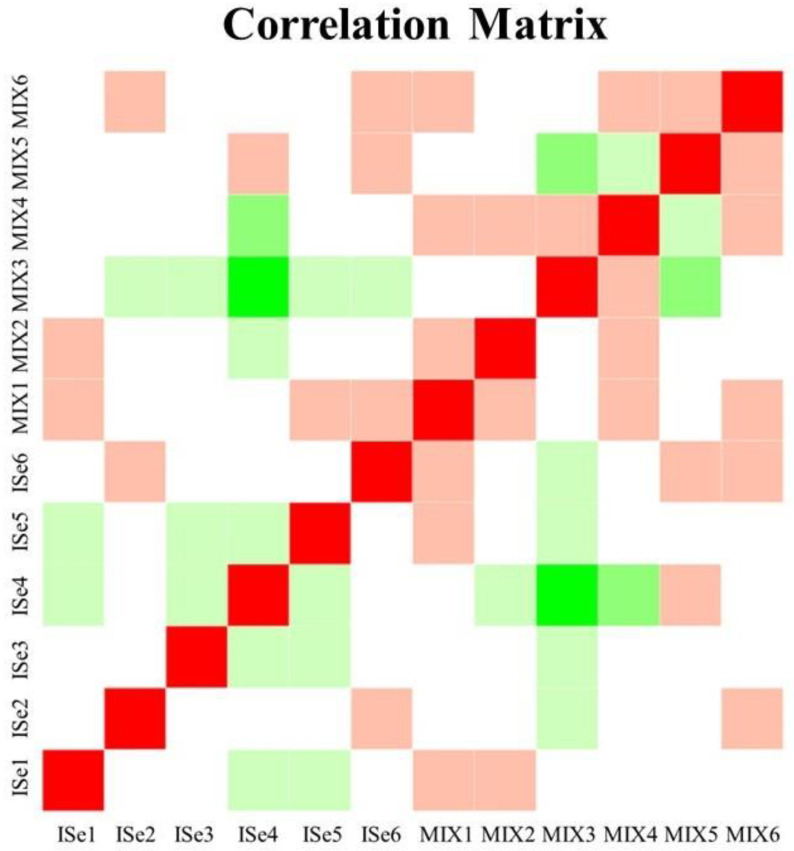
Correlation matrix of data derived from RNA-Seq analysis of each sample of caruncular (CAR) tissue from heifers supplemented with a vitamin–mineral mix containing 35 ppm as ISe (n = 6) or 1:1 mixture of ISe:OSe (MIX, n = 6). The average correlation among all samples was 0.97.

Count data were uploaded to Integrated Differential Expression and Pathway Analysis (iDEP.96, [82]), and lowly expressed transcripts were removed at <1.0 count per million (CPM). Data were log2 transformed and then subjected to principal component analysis (PCA) and hierarchical clustering of the top 1000 expressed genes to visualize sample variation. For all samples, the average mapping percentage of the identified transcripts was 92.33%. The total counts for all samples are displayed in Figure 4A, and data for each sample were normally distributed (Figure 4B). The average correlation among all samples was 0.97, as indicated in Figure 5. Differentially expressed gene/transcript (DEG) expression analysis was completed using DESeq2 v1.28.0 [83], which uses the Wald test for hypothesis testing. At the significance level *p* < 0.05, a total of 2029 gene transcripts demonstrated an effect of treatment with a false discovery rate (FDR) of <40%. Presently, the high FDR appears to be due to the small sample size, sample variation, and relatively low proportion of DEGs in the MIX compared to ISe treatment groups. However, it is generally accepted that corroborating multiple gene transcripts after high-throughput analysis by qPCR provides validation of the observed changes [84].

To assess global effects of Se treatment on the abundance of CAR transcripts at MRP, DEGs identified in DESeq2 analysis were analyzed for canonical, functional, and network analyses using QIAGEN’s Ingenuity Pathway Analysis (IPA, QIAGEN, Redwood City, CA, USA, http://www.ingenuity.com (accessed on 10/03/2023)). The raw data (FASTQ files) of this manuscript were deposited into the National Center for Biotechnology Information (NCBI) Sequence Read Archive (SRA, accession number PRJNA1029628, release date: 30 April 2024).

### 4.5. Real-Time PCR Analysis

Real-time PCR (qPCR) was used to quantify the relative abundance of mRNA in CAR samples for identified genes that differed according to the RNA-Seq analysis and to characterize mRNA transcript abundance of selenoproteins and related receptors in CAR at MRP using a technique that is routinely used in our laboratory [10,34,43]. Approximately 1 μg of RNA from each sample was reverse-transcribed into cDNA using SuperScript IV VILO Master Mix with ezDNAse Enzyme (Invitrogen by Thermo Fisher Scientific, Vilnius, Lithuania). Each sample was compared to a no-reverse-transcription control to ensure that subsequent qPCR results were not a result of genomic DNA contamination.

Real-time PCR analysis occurred in two experiments. Initially, the relative abundance of mRNAs encoding the selenoproteins DIO1, DIO2, DIO3, GPX1, GPX2, GPX3, GPX4, GPX6, TXNRD1, TXNRD2, TXNRD3, SELENOF, SELENOH, SELENOI, SELENOK, SELENOM, SELENON, SELENOO, SELENOP, SELENOR, SELENOS, SELENOT, SELENOV, SELENOW, and SEPHS2, and the selenoprotein P receptors LRP2, LRP8, and TFRC was determined.

Subsequently, the relative abundance of mRNA transcripts *Ifit2*, *Irf9*, *Isg20*, *Scara5*, *Scarb1*, *Timp2*, *Timp3*, and *Trim56* was determined to corroborate RNA-Seq results herein. Additionally, corroborating qPCR for the mRNA transcripts *Ifit3*, *Irf1*, *Isg15*, *Oas2*, and *Rsad2* were published previously in Crites et al. [34] in a targeted mRNA analysis prior to conducting RNA sequencing in the present study.

Primers were designed against each respective RefSeq using the NCBI Primer-BLAST tool (https://www.ncbi.nlm.nih.gov/tools/primer-blast/ (accessed on 9 June 2023)). Target products in cDNA were verified by DNA sequencing at ACGT, Inc. (Wheeling, IL, USA), and sequencing results were compared to each respective primer template using the NCBI Nucleotide-BLAST tool (https://blast.ncbi.nlm.nih.gov/Blast.cgi?PROGRAM=blastn&BLAST_SPEC=GeoBlast&PAGE_TYPE=BlastSearch (accessed on 8 August 2023)). The GenBank accession numbers, forward and reverse primer sequences, amplicon length of each product, and product identify for each transcript of interest are listed in Appendix A. To perform qPCR analysis, a total volume of 25 μL containing 5 μL of cDNA, 1 μL of a 10 μM stock of each primer (forward and reverse), 12.5 μL of 2 × SYBR Green PCR Master Mix (iTaq Universal SYBR Green Supermix, BIO-RAD, Hercules, CA, USA), and 5.5 μL of nuclease-free water was used. Reactions were conducted using a Bio-Rad CFX Maestro thermal cycler (Bio-Rad, Hercules, CA, USA).

The relative abundance of each transcript was quantified using the 2^−ΔΔCT^ method [85] using transcripts for three consecutively expressed normally distributed genes that were not affected by Se treatment to normalize the data. For selenoprotein and selenoprotein P mRNA analyses, *Gapdh*, *Hprt1*, and *Sdha* transcripts were used as reference genes. Alternatively, for transcriptomics corroboration mRNA analysis, *Gapdh*, *Hprt1*, and *RPS11* transcripts were the respective reference genes. Data were normalized to the expression level of ISe. A total of six heifers per treatment were analyzed via qPCR, and all reactions were performed in triplicate.

### 4.6. Statistical Analysis

Data are presented as least square means (±SEM), with individual heifer as the experimental unit. Data were analyzed for normal distribution and homogeneity. When appropriate, qPCR data were natural log transformed given that the samples were not distributed normally. For selenoprotein mRNA analysis, the transcripts *Dio2*, *Gpx2*, *Selh*, *Selr*, *Selw*, and *Tfrc* were natural log transformed, and for mRNA analysis to corroborate transcriptomic data, *Isg20* was natural log transformed due to being non-normally distributed. The relative expression for each transcript identified in RNA-sequencing results was subjected to the Wald test using DESeq2, as described in Section 4.4. “RNA-Sequencing” above.

To determine the effect of form of Se on concentrations of each mRNA transcript, data were analyzed using Student’s *t*-test with the PROC TTest procedure of SAS statistical software package (version 9.4; SAS Institute, Inc., Cary, NC, USA), and each treatment group contained six heifers (ISe, n = 6; MIX, n = 6). Results were considered statistically significant at *p* ≤ 0.05 or with a tendency to differ at 0.05 < *p* ≤ 0.10.

## 5. Conclusions

This research had two distinct objectives. Initially, we performed a targeted mRNA analysis to quantify the relative abundance of mRNA encoding selenoproteins and selenoprotein P receptors in the CAR at d 17 of gestation (MRP) in response to ISe- or MIX- supplemental Se. Subsequently, we employed a global transcriptomic approach to expand upon previous findings in our lab that identified key changes in the abundance of mRNA encoding the P4 and IFNτ-induced DGAT2, FGF2, IFIT3, ISG15, MX1, OAS2, and RSAD2 in response to MIX-form Se diet compared to ISe, which occurred synonymously with longer conceptuses in MIX-supplemented heifers [34]. Therefore, the second objective of this study was to examine transcriptomic changes using RNA sequencing analysis in the CAR tissue of the endometrium at MRP, testing the hypothesis that there will be changes in the endometrium indicating an advancement in CAR preparation for implantation of the conceptus. Results from the current transcriptomic analysis correspond with this hypothesis, as evidenced by the significant downregulation of interferon signaling through the JAK/STAT1/2 signal transduction pathway and downregulation of mRNA encoding the classical ISGs: IFIT1, IFIT2, IFIT3, IRF1, IRF9, ISG15, ISG20, OAS2, and RSAD2. This presumed shift in the timing of MRP appears plausible given the additional findings of changes in the cellular organization and function, and immune evasion occurring in MIX-supplemented heifers compared to those supplemented with ISe alone.

## Figures and Tables

**Table 1:** Relative abundance of mRNA encoding selenoproteins and selenoprotein P receptors in the caruncular (CAR) tissue of ISe- (n = 6) or MIX- (n = 6) treated heifers ^1^.

Gene ^2^	Gene Name	qPCR ^3^			
		ISe	MIX	SEM	*p*-Value ^4^
*Iodothyronine deiodinases*					
*Dio1*	Iodothyronine deiodinase 1	Unable to be detected			
*Dio2*	Iodothyronine deiodinase 2	1.15	0.53	0.25	<0.05
*Dio3*	Iodothyronine deiodinase 3	1.03	0.73	0.10	<0.05
*Glutathione peroxidases*					
*Gpx1*	Glutathione peroxidase 1	1.01	0.90	0.09	0.40
*Gpx2*	Glutathione peroxidase 2	1.41	0.91	0.36	0.68
*Gpx3*	Glutathione peroxidase 3	1.14	1.26	0.16	0.66
*Gpx4*	Glutathione peroxidase 4	1.05	1.15	0.14	0.66
*Gpx6*	Glutathione peroxidase 6	1.11	0.96	0.24	0.67
*Thioredoxin reductases*					
*Txnrd1*	Thioredoxin reductase 1	1.08	1.00	0.15	0.69
*Txnrd2*	Thioredoxin reductase 2	1.04	0.99	0.11	0.75
*Txnrd3*	Thioredoxin reductase 3	1.02	0.95	0.09	0.58
*Other selenoproteins*					
*Selenof*	Selenoprotein F	1.05	1.15	0.15	0.66
*Selenoh*	Selenoprotein H	1.06	0.79	0.12	0.18
*Selenoi*	Selenoprotein I	1.01	1.02	0.07	0.97
*Selenok*	Selenoprotein K	1.03	1.12	0.12	0.61
*Selenom*	Selenoprotein M	6.59	5.36	2.90	0.77
*Selenon*	Selenoprotein N	1.01	0.96	0.07	0.56
*Selenoo*	Selenoprotein O	1.05	1.12	1.10	0.63
*Selenop*	Selenoprotein P	1.04	1.09	1.10	0.71
*Selenor*	Selenoprotein R	1.11	1.11	0.18	0.68
*Selenos*	Selenoprotein S	1.07	1.11	0.14	0.85
*Selenot*	Selenoprotein T	1.01	1.03	0.06	0.84
*Selenov*	Selenoprotein V	1.10	1.14	0.21	0.90
*Selenow*	Selenoprotein W	1.02	0.93	0.09	0.47
*Selenophosphate synthetase*					
*Sephs2*	Selenophosphate synthetase 2	1.02	1.15	0.08	0.25
*Selenoprotein P receptors*					
*Lrp2*	LDL receptor related protein 2	1.02	0.78	0.11	0.17
*Lrp8*	LDL receptor related protein 8	1.05	0.85	0.11	0.26
*Tfrc*	Transferrin receptor	1.10	0.93	0.17	0.62

^1^ Selenium was supplemented at 35 ppm as either inorganic (ISe; sodium selenite) or a 1:1 combination (MIX) of ISe and OSe (1:1 sodium selenite and SEL-PLEX). Selenium was supplemented to treatment groups ad libitum. ^2^ Data for *Dio2*, *Gpx2*, *Selh*, *Selr*, *Selw*, and *Tfrc* were natural log transformed due to having not-normal distribution. ^3^ Data are expressed as a ratio of MIX relative to ISe. ^4^ *p*-values are associated with Student’s *t*-test using the PROC TTEST procedure of SAS statistical software package (version 9.4, SAS Institute Inc., Cary, NC, USA) at n = 6 per treatment.

**Table 2:** Top 10 most highly up- and downregulated differentially expressed genes (DEGs) in the caruncular (CAR) tissue of ISe- (n = 6) versus MIX- (n = 6) treated heifers ^1^.

Gene ID	Gene Description	Fold Change	*p*-Value ^2^
*Upregulated in MIX*			
CCDC152	Coiled-coil domain containing 152	4.03	0.0111
CD79B	CD79b molecule	3.75	0.0329
TM4SF5	Transmembrane 4 L six family member 5	3.39	0.0366
C22orf31	Chromosome 22 open reading frame 31	3.38	0.0152
SLC17A7	Solute carrier family 17 member 7	3.34	0.0338
CCL21	C-C motif chemokine ligand 21	2.93	0.0359
FAM180B	Family with sequence similarity 180 member B	2.88	0.0103
COL17A1	Collagen type XVII alpha 1 chain	2.35	0.0019
CCR9	C–C motif chemokine receptor 9	2.34	0.0184
OMG	Oligodendrocyte myelin glycoprotein	2.33	0.0429
*Downregulated in MIX*			
DPYSL4	Dihydropyrimidinase-like 4	−4.07	0.0108
KLK5	Kallikrein-related peptidase 5	−4.03	0.0004
GRM3	Glutamate metabotropic receptor 3	−3.70	0.0004
NIPAL4	NIPA-like domain containing 4	−3.59	0.0020
NALCN	Sodium leak channel, nonselective	−3.26	0.0134
PTGER1	Prostaglandin E receptor 1	−3.16	0.0178
COL28A1	Collagen type XXVIII alpha 1 chain	−3.12	0.0105
SLC26A8	Solute carrier family 26 member 8	−3.12	0.0026
SLC34A3	Solute carrier family 34 member 3	−3.11	0.0237
DMKN	Dermokine	−2.93	<0.0001

^1^ Selenium was supplemented at 35 ppm as either inorganic (ISe; sodium selenite) or a 1:1 combination (MIX) of ISe and OSe (1:1 sodium selenite and SEL-PLEX). Selenium was supplemented to treatment groups ad libitum. ^2^ For statistical analysis, *p*-values were determined using the Wald test in DESeq2.

**Table 3:** Top 5 IPA-identified canonical pathways of genes differentially expressed from caruncular (CAR) tissue of ISe- (n = 6) versus MIX- (n = 6) supplemented heifers ^1^.

Canonical Pathway ^2^	Gene Symbols	Ratio ^3^	Z-Score	*p*-Value ^4^
ATM Signaling	**Up**: ATR, CBX1, CBX3, CBX5, CCNB2, CDK2, HP1BP3, MDC1, PPP2R1B, RAD50, RAD51, RBBP8, RNF8, SMC2, SMC3, SMC1A, ZNF420 **Down**: BID, BRAT1, MAPK12, MAPK13, PPM1L, PPP2R1A, TP73, TRRAP	0.25 (25/100)	0.471	<0.0001
Spliceosomal Cycle	**UP**: BCAS2, CDC5L, CTNNBL1, CWC15, DDX23, DHX38, ISY1-RAB43, MAGOHB, RBM8A, SLU7, SNRNP200, ZNF830 **Down**: PRPF19, SF3B3	0.29 (14/49)	2.673	<0.0001
Role of PKR in Interferon Induction and Antiviral Response	**Up**: APAF1, DNAJC3, HMGB1, HSP90AA1, HSP90AB1, HSP90B1, HSPA4, METAP2, MSR1, REL **Down**: ATF3, BID, FADD, FOS, HSPA6, IRF1, IRF9, MAPK12, MAPK13, PDGFRB, PYCARD, RELA, STAT2, STAT3, TARBP2, TNFRSF1A	0.19 (26/136)	−3.128	<0.0001
CLEAR Signaling Pathway	**Up**: ATP6V1C1, ATP6V1E1, BECN1, BMPR1B, HPS5, ITPR2, MAP4K3, NRBF2, PPP2R1B, RRAGB, VPS26A, YWHAE **Down**: ATP6V0A1, ATP6V0D1, BLOC1S3, BMP6, CRTC2, CTNS, CTSA, FLT1, GBA1, GLB1, KCNIP3, MAPK7, MAPK12, MAPK13, MLST8, PDGFRB, PML, PPM1L, PPP2R1A, PRKAG1, PRKCB, RAB7A, RPTOR, SEC13, SESN2, TCIRG1, TGFA, TGFBR3, TNFRSF1A, TRPM1, TSC2	0.15 (43/285)	−0.762	<0.0001
Autophagy	**Up**: ATG3, ATG10, ATR, BECN1, CALM1, CDKN1B, GNAI3, NRBF2, PIK3C2A, PPP2R1B, RB1CC1, SESN1, STX17, VPS41 **Down**: ATG2A, BMP6, FOS, IRS1, IRS2, MAPK12, MLST8, PI4K2A, PPM1L, PPP2R1A, PRKAG1, RAB7A, RAB7B, RIPK1, RPTOR, SLC7A5, TGFA, TNFRSF1A, TSC2, ULK1, WIPI2	0.16 35/216	0.845	<0.0001

^1^ Selenium was supplemented at 35 ppm as either inorganic (ISe; sodium selenite) or a 1:1 combination (MIX) of ISe and OSe (1:1 sodium selenite and SEL-PLEX). Selenium was supplemented to treatment groups ad libitum. ^2^ Results from IPA were obtained based on log2 fold changes calculated using DESeq2. ^3^ The ratio calculated as the number of differentially expressed genes (*p* < 0.05) in a given pathway divided by the total number of genes that make up that pathway. ^4^ *p*-values were determined using IPA.

**Table 4:** Top 5 IPA-identified molecular and cellular functions in caruncular (CAR) tissue of ISe- (n = 6) versus MIX- (n = 6) supplemented heifers ^1^.

Molecular and Cellular Functions ^2^	Z-Score ^3^	*p*-Value ^4^
**Cell death and survival (732 molecules)**		
Necrosis	−1.392	<0.0001
Apoptosis	−2.252	<0.0001
Cell death of tumor cell lines	−1.757	<0.0001
Cell survival	1.029	<0.0001
Apoptosis of tumor cell lines	−1.425	<0.0001
Cell viability	1.112	<0.0001
Cell viability of tumor cell lines	1.278	<0.0001
Necrosis of epithelial tissue	−1.682	<0.0001
Cell death of osteosarcoma cells	−1.671	<0.0001
Cell death of bone cancer cell lines	−0.434	<0.0001
Cell death of blood cells	−1.469	<0.0001
Cell death of sarcoma cell lines	−0.943	<0.0001
Colony survival of tumor cell lines	1.183	<0.0001
Necrosis of tumor	−1.881	<0.0001
Cell death of breast cancer cell lines	−2.267	<0.0001
Apoptosis of peritoneal macrophages	−0.574	<0.0001
**Cellular response to therapeutics (149 molecules)**		
Radiosensitivity of cells	−1.38	<0.0001
**Gene expression (462 molecules)**		
Expression of RNA	−3.621	<0.0001
Transcription of RNA	−2.971	<0.0001
Transcription	−2.877	<0.0001
Transcription of DNA	−2.485	<0.0001
Activation of DNA endogenous promoter	−2.373	<0.0001
Transactivation	1.115	<0.0001
Transactivation of RNA	0.804	<0.0001
Repression of RNA	0.646	<0.0001
**RNA post-translational modification (92 molecules)**		
Processing of RNA	−1.066	<0.0001
Processing of mRNA	−1.635	<0.0001
Splicing of RNA	−1.339	<0.0001
Splicing of mRNA	−1.682	<0.0001
**Cellular assembly and organization (505 molecules)**		
Development of cytoplasm	−1.738	<0.0001
Cohesion of sister chromatids	0.854	<0.0001
Formation of centriole	0.921	<0.0001
Remodeling of chromatin	0.900	<0.0001
Formation of cellular protrusions	−0.647	<0.0001
Replication of centriole	0.921	<0.0001

^1^ Selenium was supplemented at 35 ppm as either inorganic (ISe; sodium selenite) or a 1:1 combination (MIX) of ISe and OSe (1:1 sodium selenite and SEL-PLEX). Selenium was supplemented to treatment groups ad libitum. ^2^ Results from IPA were obtained based on log2 fold changes calculated using DESeq2. ^3^ Z-score was calculated using IPA and was used to infer activation state. Only fractions resulting in a z-score with an absolute value of ≥0.5 are reported herein. ^4^ *p*-values were determined using IPA.

**Table 5:** RNA-Seq corroboration of select interferon responsive transcripts at MRP from caruncular (CAR) tissue of ISe- (n = 6) or MIX- (n = 6) supplemented heifers ^1^.

		RNA-Seq ^3^			qPCR ^3^			
Gene ^2^	Gene Name	ISe	MIX	*p*-Value	ISe	MIX	SEM	*p*-Value ^4^
*Ido1*	Indoleamine 2,3-dioxygenase 1	1.00	0.68	0.02	Not corroborated			
*Ifit1*	Interferon-induced protein with tetratricopeptide repeats 1	1.00	0.83	0.04	Not corroborated			
*Ifit2*	Interferon induced protein with tetratricopeptide repeats 2	1.00	0.71	0.01	1.11	0.54	0.17	0.04
Ifit3 *	Interferon induced protein with tetratricopeptide repeats 3	1.00	0.78	0.02	1.08	0.65	0.13	0.04
Irf1 *	Interferon regulatory factor 1	1.00	0.79	0.02	1.05	0.75	0.11	0.08
*Irf9*	Interferon regulatory factor 9	1.00	0.83	<0.01	1.01	0.95	0.06	0.47
Isg15 *	ISG15 ubiquitin like modifier	1.00	0.78	<0.01	1.04	0.76	0.09	0.05
*Isg20*	Interferon stimulated exonuclease, transcript variant 1	1.00	0.74	<0.01	1.06	0.76	0.12	0.11
Oas2 *	2′-5′-oligoadenylate synthetase 2	1.00	0.81	0.01	1.03	0.67	0.10	0.01
*Rsad1*	Radical S-adenosyl methionine domain containing 1	1.00	0.83	<0.01	Not corroborated			
Rsad2 *	Radical S-adenosyl methionine domain containing 2	1.00	0.75	<0.01	1.05	0.57	0.14	0.01
*Scara5*	Scavenger receptor class A member 5	1.00	0.87	0.09	1.04	0.71	0.09	0.02
*Scarb1*	Scavenger receptor class B member 1	1.00	0.83	0.01	1.04	0.81	0.09	0.12
*Stat1*	Signal transducer and activator of transcription 1	1.00	0.92	0.16	Not corroborated			
*Stat2*	Signal transducer and activator of transcription 2	1.00	0.84	0.02	Not corroborated			
*Tgfb1*	Transforming growth factor beta 1	1.00	0.87	0.06	Not corroborated			
*Timp2*	TIMP metallopeptidase inhibitor 2	1.00	0.86	0.26	1.02	0.93	0.08	0.48
*Timp3*	TIMP metallopeptidase inhibitor 3	1.00	0.83	<0.01	1.02	0.79	0.08	0.06
*Trim56*	Tripartite motif containing 56	1.00	0.83	0.02	1.04	0.55	0.09	<0.01

^1^ Selenium was supplemented at 35 ppm as either inorganic (ISe; sodium selenite) or a 1:1 combination (MIX) of ISe and OSe (1:1 sodium selenite and SEL-PLEX). Selenium was supplemented to treatment groups ad libitum. ^2^ qPCR data for *Isg20* were natural log transformed due to having not-normal distribution. ^3^ Data are expressed as a ratio of MIX relative to ISe, and *p*-values are associated with statistical significance between treatment groups for each respective test. ^4^ *p*-values were obtained from Student’s *t*-test using the PROC TTEST procedure of SAS statistical software package (version 9.4; SAS Institute, Inc., Cary, NC, USA) at n = 6 per treatment. * qPCR results are reported in [34].

## Data Availability

The raw data (FASTQ files) are available in the National Center for Biotechnology Information (NCBI) Sequence Read Archive (SRA, accession number PRJNA1029628).

## Appendix A

**Table A1:** Primer sets and product identities of qPCR analysis of selenoprotein and selenoprotein P transcripts.

Gene	Gene Name	Accession Number ^1^	Oligonucleotide Primer Design (5′ to 3′) Direction	Amplicon Length (bp)	Product Identity (%) ^2^
Enzymatic transcripts					
*Dio1*	Iodothyronine deiodinase 1	NM_001122593.2	F: TCCTGTAGTCCGCCTGTCA R: TCCGGTGATTCTTGATGTCCA	242	99
*Dio2*	Iodothyronine deiodinase 2	NM_001010992.4	F: GATGGGCATCCTCAGCGTAG R: TTCTCCTGGGCACCATTTCC	315	100
*Dio3*	Iodothyronine deiodinase 3	NM_001010993.3	F: AAGTGGAGCTCAACAGCGAT R: AGTCGAGGATGTGCTGGTTC	213	100
Glutathione peroxidases					
*Gpx1*	Glutathione peroxidase 1	NM_174076.3	F: GCAACCAGTTTGGGCATCAG R: TAGGGTCGGTCATGAGAGCA	210	100
*Gpx2*	Glutathione peroxidase 2	NM_001163139.2	F: AACAGCCTCAAGTACGTCCG R: TCGGTCATGAGGGAAAACGG	158	100
*Gpx3*	Glutathione peroxidase 3	NM_174077.5	F: GCACCATCTATGAGTACGGGG R: CCCCATTCACATCGCCTTTC	315	100
*Gpx4*	Glutathione peroxidase 4	NM_174770.3	F: GATCAAAGAGTTCGCCGCTG R: CCATACCGCTTCACCACACA	198	100
*Gpx6*	Glutathione peroxidase 6	NM_001163142.1	F: CACTGTTCCTGGTCGGCTTA R: CCCAGCACAACTACACCGAA	259	100
Thioredoxin reductases					
*Txnrd1*	Thioredoxin reductase 1	NM_174625.5	F: AAGGCCGCGTTATTTGGGTA R: CCTGGTGTCCCTGCTTCAAT	306	100
*Txnrd2*	Thioredoxin reductase 2	NM_174626.2	F: CAAATGGCTTCGCTGGTCAC R: TTCGTATGCACACCAGCCTT	230	100
*Txnrd3*	Thioredoxin reductase 3	XM_015468824.1	F: CGGCGTATGACTACGACCTC R: GACTGTACTCCCAGCCGAAC	249	100
Other selenoproteins					
*Selenof*	Selenoprotein F	NM_001034759.2	F: GCAGCTCCTGTGATTTGCTT R: TTTAGCACAGGGTCTGAACCG	241	100
*Selenoh*	Selenoprotein H	NM_001321327.1	F: CACGAGCTGACGAGTCTACG R: CTTCTTCAGCTCCTCCAGCA	235	100
*Selenoi*	Selenoprotein I	NM_001075257.2	F: TCTGGCTTTCTGCTGGTTGT R: TGGTCAAAAAGCTCCCCCAG	212	100
*Selenok*	Selenoprotein K	NM_001037489.3	F: CCGTTTTGTCGATTCACGGC R: CAGATGAGCTTCCGTAGCCT	278	100
*Selenom*	Selenoprotein M	NM_001163171.2	F: CCCACTCTACCACAACCTGG R: ACCTAAAGGTCTGCGTGGTC	249	100
*Selenon*	Selenoprotein N	NM_001114976.2	F: GTGGCCATGTACCCCTTCAA R: GGGATGGGTTCTCCTGGTTG	265	100
*Selenoo*	Selenoprotein O	NM_001163193.2	F: TGGACAGGTATGACCCCGAT R: ATCTTCTGCAGGTAGTGCCG	202	100
*Selenop*	Selenoprotein P	NM_174459.3	F: TCAGGTCTTCATCACCACCA R: GTGGCAACAGCAGCTACTCA	201	100
*Selenor*	Selenoprotein R, Methionine sulfoxide reductase B1 (MSRB1)	NM_001034810.2	F: GAACCACTTTGAGCCGGGTA R: GGCCATCGTTCAGGAACTCA	221	100
*Selenos*	Selenoprotein S	NM_001046114.3	F: CCCACCCTCGAGACCGA R: GCCCAGGACTGTCTTCTTCC	394	100
*Selenot*	Selenoprotein T	NM_001103103.2	F: TGGTCACCTTCCATCCATGC R: AAGAGGTACAACGAGCCTGC	240	100
*Selenov*	Selenoprotein V	NM_001163244.2	F: ACTCCATTGGCCACCGATTT R: AGGCCACAGTAAACCACTCG	224	100
*Selenow*	Selenoprotein W	NM_001163225.1	F: AGTGTTCGTAGCGGGAAAGC R: CGCGAGAACATCAGGGAAGG	233	98
Selenophosphate synthetase					
*Sephs2*	Selenophosphate synthetase 2	NM_001114732.2	F: GATCCCTACATGATGGGGCG R: GTTTACCACCGTTTGCCCAC	219	100
Selenoprotein P receptors					
*Lrp2*	LDL receptor related protein 2	XM_024983502.1	F: GTGGTTTGGGTTACCGTTGC R: GGCACCCTGTTAGCTGTGAT	304	99
*Lrp8*	LDL receptor related protein 8	NM_001097565.1	F: AGCCACCCTTTTGGGATAGC R: AAGGCACAGGTACTCACAGC	231	100
*Tfrc*	Transferrin receptor	NM_001206577.1	F: CCAGGTTTAGTCTGGCTCGG R: GGTCTGCCCAGAATATGCGA	339	99

^1^ These contents are associated with each gene symbol and are the accession numbers of the sequences retrieved from the NCBI RefSeq database for designing primers and probes. ^2^ All qPCR products were validated by sequencing. The identity values (%) presented are the base pair ratios between the total amplicon length and the number of identical base pairs.

**Table A2:** Primer sets and product identities of qPCR analysis of IFNτ-responsive transcripts and all reference transcripts.

Gene	Gene Name	Accession Number ^1^	Primer Design (5′ to 3′) Direction	Amplicon Length (bp)	Product Identity (%) ^2^
*Ifit2*	Interferon induced protein with tetratricopeptide repeats 2	XM_002698356.5	F: CAGATGTGATTCGAGGGGCA R: CATGGAGGCAGGCGAGATAG	282	100
*Ifit3 **	Interferon induced protein with tetratricopeptide repeats 3	NM_001075414.1	F: ATTCTGAAGCAGGCCGTTGA R: TCCAGTGCCCTTAGCAACAG	224	100
*Irf1 **	Interferon regulatory factor 1	NM_001191261.2	F: ACAGCCCCGATACCTTCTCT R: CTTCCCATCCACGCTTGTCT	338	100
*Irf9*	Interferon regulatory factor 9	NM_001024506.1	F: GCGCTGTGCTCTCAACAAAA R: AAGTCTAAACGGCCAGCTCC	285	100
*Isg15 **	ISG15 ubiquitin like modifier	NM_174366.1	F: CCATCCTGGTGAGGAACGAC R: GAACACGGTGCACCCCTTCA	200	99
*Isg20*	Interferon stimulated exonuclease gene 20	XM_002696514.5	F: CTTGTGGACTACCACGGCTC R: GATGGCGTAGTCGCTCATGT	234	98
*Oas2 **	2′-5′-oligoadenylate synthetase 2	NM_001024557.1	F: ACTGGTTTCAAAAGTGCCAGG R: CAGCCAGCAGGTGTTATCCA	314	98
*Rsad2 **	Radical S-adenosyl methionine domain containing 2	NM_001045941.1	F: GTGGTTCCAGAAGTACGGTGA R: AACCGTTCCGCTTCTCTCAG	315	100
*Scara5*	Scavenger receptor class A member 5	NM_001102499.1	F: AGGACCTACGCCTCAAGGAT R: GGGCCTCGATCACCTTTGAA	256	100
*Scarb1*	Scavenger receptor class B member 1	NM_174597.2	F: GCAGACATGGGCAACCTCT R: GCCTTGGATGATCCCCTCAG	249	100
*Timp2 ^ⱡ^*	TIMP metallopeptidase inhibitor 2	NM_174472.4	F: GGGTCTCGCTGGACATTG R: TTGATGTTCTTCTCCGTGACC	256	100
*Timp3*	Bos taurus TIMP metallopeptidase inhibitor 3 (TIMP3), mRNA	NM_174473.4	F: GGATTCACCAAGATGCCCCA R: GCAGTTACAGCCCAGGTGAT	222	99
*Trim56*	Tripartite motif containing 56	NM_001206574.1	F: TTCAGACCCCAAATCAGGAC R: TCTGGGCTCTGCTCTCTTTC	126	99
Housekeeping Transcripts					
*Gapdh*	Glyceraldehyde 3-phosphate dehydrogenase	NM_001034034.2	F: ACATCAAGTGGGGTGATGCT R: GGCATTGCTGACAATCTTGA	200	99
*Hprt1*	Hypoxanthine phosphoribosyltransferase 1	NM_001034035.2	F: GCCAGCCGGCTACGTTAT R: ATCCAACAGGTCGGCAAAGA	256	100
*Rps11*	Ribosomal protein S11	NM_001024568.2	F: AAGATGGCGGACATTCAGAC R: GCCCTCGAATGGAGACATTA	214	99
*Sdha*	Succinate dehydrogenase complex flavoprotein subunit A	NM_174178.2	F: GCAGAACCTGATGCTTTGTG R: CGTAGGAGAGCGTGTGCTT	185	99

^1^ These contents are associated with each gene symbol and are the accession numbers of the sequences retrieved from the NCBI RefSeq database for designing primers and probes. ^2^ All qPCR products were validated by sequencing. The identity values (%) presented are the base pair ratios between the total amplicon length and the number of identical base pairs. * Primers are reported in [34]. ^ⱡ^ Primer reported in [86].
